# Traceability of Plant Diet Contents in Raw Cow Milk Samples

**DOI:** 10.3390/nu1020251

**Published:** 2009-12-04

**Authors:** Elena Ponzoni, Francesco Mastromauro, Silvia Gianì, Diego Breviario

**Affiliations:** Institute of Agricultural Biology and Biotechnology (IBBA), Italian National Research Council (CNR), Milan 20133, Italy; Email: mastromauro@ibba.cnr.it (F.M.); giani@ibba.cnr.it (S.G.); terenzio@ibba.cnr.it (D.B.)

**Keywords:** DNA detection, traceability, feed

## Abstract

The use of molecular marker in the dairy sector is gaining large acceptance as a reliable diagnostic approach for food authenticity and traceability. Using a PCR approach, the *rbcL* marker, a chloroplast-based gene, was selected to amplify plant DNA fragments in raw cow milk samples collected from stock farms or bought on the Italian market. *rbcL*-specific DNA fragments could be found in total milk, as well as in the skimmed and the cream fractions. When the PCR amplified fragments were sent to sequence, the nucleotide composition of the chromatogram reflected the multiple contents of the polyphytic diet.

## 1. Introduction

Requirements for traceability/product tracing have been introduced in national regulations as an effective means to contribute to product safety and consumer confidence. Under EU law, “traceability” means the ability to track any food, feed, food-producing animal or substance that will be used for consumption, through all stages of production, processing and distribution [1]. In particular the dairy sector has taken “the viewpoint that traceability/product tracing as a tool is not only relevant in the context of food inspection and certification systems, but may also be applied for industry-driven business reasons and is mainly concerned with food safety aspects” (Bulletin of the International Dairy Federation 412/2007; IDF Guiding Principles for Traceability/Product Tracing). The ability to confirm the authenticity of traded foods is vital to improve the integrity of the food supply chain. Using a variety of advanced chemical, biochemical and molecular biological techniques many laboratories are now able to assess the provenance of food and feed [2]. In particular, molecular markers are becoming an useful tool to trace the whole chain: they provide a diagnostic approach for food authenticity that can benefit the farmers, the dairy industries and the traders. Species-specific PCR has shown to be a suitable method to control food authenticity because a specific target sequence can be detected even in matrices containing a pool of heterogeneous genomic DNA, such as milk or other dairy products [3,4]. Recently, our research group has successfully applied newly developed molecular markers of both nuclear and plastidial origin to the molecular characterization of different grass species and forages commonly fed to animals [5]. These results led us to investigate, with a PCR-based approach, about the possibility that molecular traces resulting from the feed could also be tracked in milk samples, as the end product of the whole dairy chain that starts from the animal diet. Food-ingested foreign DNA in fact is not completely degraded in the gastrointestinal tract. Research on the fate of foreign DNA in mammalian organism showed that foreign DNA could be detected therein by PCR specific amplification. It was concluded that DNA fragments could reach the bloodstream because the gastrointestinal tract is not an absolute barrier against the uptake of macromolecules and that foreign DNA can be transported through the epithelium of the gut and the cells of the Peyer’s patches to cells of spleen and liver [6,7]. In addition, there is some evidence that fragments of foreign DNA might enter the organism or become incorporated into cells lining the gut wall [8]. Previous studies with *Bt* corn showed that plant DNA fragments of different lengths could be associated with organs and muscles in beef, pork, and poultry [9,10]. Recently, a couple of laboratories have reported the presence of short fragments of plant DNA in cow milk [11,12]. The aim of our studies was to further confirm those findings and to demonstrate that DNA sequencing can be used to identify the plant species present in the feed supplied to animals. To this purpose, raw cow milk samples fetched from stock farms or sold in the Italian market were investigated.

## 2. Results and Discussion

### 2.1. Optimization of Plant DNA Extraction in Milk

Ordinarily, the DNA sources investigated and found in milk are those of animal [13] or bacterial origin [14]. The aim of this study was instead the search of traces of plant DNA derived from the diet fed to the animals. Since the success of this search could possibly depend on the method of extraction, we compared yield and purity of total DNA obtained by three different protocols applied on the same raw cow milk sample, a brand-labeled milk commonly sold in the Italian market.

DNA yield and purity were checked by measuring the A_260_/A_280_ absorbance ratio. These data are summarized in Table 1. None of the extraction protocol allowed the isolation of a highly pure total DNA fraction since the A_260_/A_280_ ratio value was always lower than 1.8. This typically occurs because of protein contamination resulting from micelles, stable protein complexes finely dispersed in the water phase of milk.

**Table 1:** Comparison of three different DNA extraction protocols performed on the same brand-labeled raw cow milk sample. The quality of total DNA is expressed by the A_260_/A_280_ ratio value. The total DNA yield is calculated per mL of extracted milk sample or fraction of it and represents the average of at least two different DNA extractions.

DNA extraction method used	Starting material	DNA quality A_260_/A_280_	DNA yield/mL of milk
CTAB-NaCl buffer protocol	20 mL	0.98	190 μg
CTAB/phenol:chloroform protocol	20 mL	1.5	950 μg
Hexane protocol skim fraction	17 mL	1.4	460 μg
Hexane protocol fat fraction	3 mL	1.3	620 μg

As reported in Table 1, considerably higher yields of total milk DNA were obtained with the CTAB/phenol:chloroform extraction method. However, even when this method was used, a variability in the final yield occurring from sample to sample was frequently observed. High yield in total DNA is essentially contributed by the genomic DNA released from somatic animal cells, that amount to SCC of >100,000 cells/mL [16]. Even so, larger amounts of total DNA are more likely to carry a higher proportion of DNA fragments of the diet. Therefore, a combination of high yield and purity of total DNA was assumed as an important preliminary requisite for the detection of DNA sequences of plant origin. Such a criteria is certainly not met by the CTAB-NaCl buffer protocol that in fact was discarded. 

**Figure 1:**
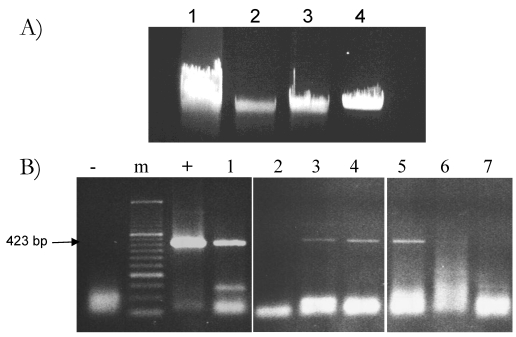
**(A)** Total DNA extracted from whole milk (lane 1), skimmed (lane 2) and fat (lane 3) milk fractions extracted by CTAB/phenol:chloroformprotocol. Milk-derived DNA samples are compared to a known amount of standard high molecular weight phage λ DNA (lane 4). **(B)**. *rbcL* 423 bp long fragment, PCR amplified with the Lrub fw/rv primer combination from the whole, skimmed and fat milk DNA samples shown in panel A, used at the following amount: lane 1: 400 ng of total milk DNA; lane 2, 3, 4: 200 ng, 400 ng and 800 ng of DNA recovered from the skimmed fraction; lane 5, 6, 7: 200 ng, 400 ng and 800 ng of DNA recovered from the fat fraction; m: molecular marker; “-”: negative control (just buffer); “+”: plant DNA positive control.

Since the free DNA dispersed in milk, where fragments of plant origin are presumably present, is mainly concentrated in the cream fraction [17], a hexane-based preparation protocol was also performed. This allows the separation of the skimmed and the fat-enriched milk fractions before DNA extraction. The fat fraction is removed from the surface of the milk sample by a brief centrifugation step (for details see Table 1 and the Experimental section). The left over is the skimmed fraction. Figure 1A shows the results of one of such experiments: although total DNA distributes in both fractions, significant amounts of it are clearly adsorbed by fat. Routinely, the amount of total DNA we recovered from the fat fraction was 8- to 10-fold higher than that obtained from the skimmed fraction, when normalizing for the different starting volumes and the yield. The sensitivity range of plant DNA detection in total DNA samples obtained from the CTAB/phenol:chloroform protocol, combined or not with the hexane-based preparation step, was also evaluated. 

We selected, as the target sequence for plant DNA detection, the chloroplast-located gene that encodes for the large subunit of rubisco (ribulose bisphosphate carboxylase gene, *rbcL*), the most abundant plant enzyme involved in the first step of the dark phase of photosynthesis. Sequences comparison and multiple alignment of different plant *rbcL* genes available in the GenBank/EMBL database allowed the design of two sets of primers: Arub fw/rv and Lrub fw/rv that amplify a 348 bp and 423 bp long fragment respectively. These sets of primers could successfully detect plant DNA fragments in goat milk samples [15]. The RUB-F2/R2 couple of primers, capable of amplifying a 351 bp long fragment, was also used [11].

The rubisco PCR assay was performed on the DNA extracted from either the skimmed or the fat-enriched fraction of milk. For each fraction, three different amounts of total DNA (200 ng, 400 ng and 800 ng) were used to amplify the 423 bp *rbcL* fragment with the Lrub fw/rv set of primers. The level of amplification was compared with that obtained on 400 ng of total DNA extracted, by the standard CTAB/phenol:chloroform procedure, from the whole milk sample. As shown in Figure 1B, detection of the *rbcL* fragment in the skimmed fraction was consistently successful when using the two higher amounts of DNA (400 ng and 800 ng). Occasionally, under some specific experimental conditions, *rbcL* PCR-amplified fragments could also be detected when using 200 ng of total DNA amount. On the opposite, amplification of the *rbcL* fragment on DNA extracted from the fat fraction was commonly obtained when using 200 ng amount. Likely, this discrepancy is due to the presence of a large amount of PCR inhibitors in the fat fraction of milk. Therefore, when we increase the amount of DNA per reaction we also increase the level of inhibitors. On the contrary, detection of the target *rbcL* sequence in the skimmed fraction, presumably poorer in inhibitors, is favored by higher amount of total DNA, allowing for a better amplification of trace amounts of plant plastidial DNA. Overall, these data indicate that a successful detection of DNA of plant origin in milk depends on a critical balance between the amount of target DNA sequences and that of PCR inhibitors. For this reason we have routinely worked with the skimmed fraction of milk to raise our chance of detection of plant DNA fragments 

### 2.2. Feed-Derived Plant DNA Detection in Different Milk Samples

**Figure 2:**
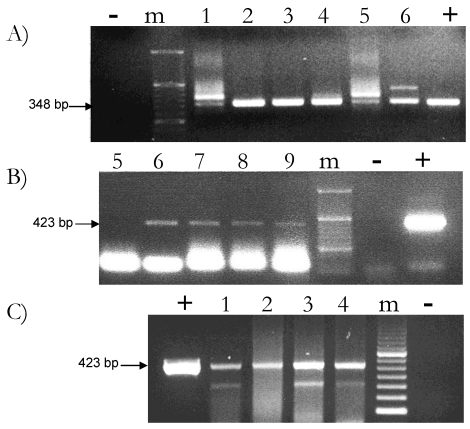
PCR-mediated detection of rubisco DNA fragments in nine different raw cow milk samples collected from stock farms (A-B) and in two different brand-labeled milk samples sold in the Italian market (C). **(A)** *rbcL* 348 bp long fragment amplified with the use of the Arub fw/rv set of primers from 400 ng of DNA isolated from the skimmed fraction of six different cow milk samples. **(B)** *rbcL* 423 bp long fragment amplified with the use of the Lrub fw/rv set of primers from 400 ng of DNA isolated from the skimmed fraction of five different cow milk samples. Milk samples number 5 and 6 are in common between the two panels. **(C)** *rbcL* 423 bp long fragment amplified with the use of the Lrub fw/rv set of primers from 2 brand labeled milk samples. Lane 1, 2: 400 ng and 800 ng of total DNA extracted from n° 1 brand-labeled sample; lane 3, 4: 400 ng and 800 ng of total DNA extracted from n° 2 brand-labeled sample; m: molecular marker; “+”: plant DNA positive control. “-”: negative PCR control (just buffer, no DNA).

The presence of plant DNA was assessed in several cow milk samples by PCR amplification of a *rbcL* fragment. Since cow milk is a complex food matrix, the composition of which may reflect differences in animal species, breed, stage of lactation and feed composition, different cow milk samples collected from stock farms or bought in the market were analyzed. Rubisco gene-specific analyses were carried out with two different PCR assays. Figure 2 reports some of these results. 

For each reaction, 400 ng amount of DNA obtained from the skimmed fraction of milk was used to amplify the *rbcL* fragment with either the Arub fw/rv (Figure 2A) or the Lrub fw/rv (Figure 2B) set of primers. Samples n° 5 and n° 6 were actually tested with both. Figure 2C shows the amplification of the 423 bp *rbcL* gene fragment in two different brand-labeled raw cow milk samples commonly sold in the North Italy market. In this case, PCR-based amplification was performed with the use of the Lrub fw/rv set of primers on two different amounts, 400 ng and 800 ng, of total DNA extracted from the skimmed fraction of milk. 

The combinatorial use of the two different, *rbcL*-specific sets of primers, capable of amplifying fragments of different length, was conceived to increase the chance of detection of plant DNA. In fact, it has been already reported that the lower are the sizes of the amplified fragment the higher is the chance of detection since the DNA ingested with the diet undergoes extensive degradation and smaller size fragments, less than 500 bp long, are prevalent than larger. This seems to be the case of milk sample n° 5, only detected when using the Arub fw/rv couple of primers (Figure 2). The specificity of the primers, not only the sizes of the amplified fragment, could also be an important factor for detection and may determine differences in the yield of amplification. Even so, we have been able to concomitantly detect *rbcL* fragments of three different sizes (348 bp, 351 bp and 423 bp long, respectively) in the vast majority of the cow milk samples we have analyzed. Amplification of these fragments was successful in 70% of samples that is a percentage of detection closed to that previously reported for similar experiments [12]. This percentage of detection may either reflect an unequal distribution of the plant DNA fragments in the different milk samples or may depend from a different amount of PCR inhibitors. Increasing the percentage of plant DNA detection in raw cow milk samples represents one of the future experimental challenges. Our data, reporting for the first time the consistent amplification of DNA of plant origin from commercially sold milk samples, may help in this achievement.

### 2.3. Reproducibility of Plant DNA Detection in Cow Milk Samples

**Figure 3:**
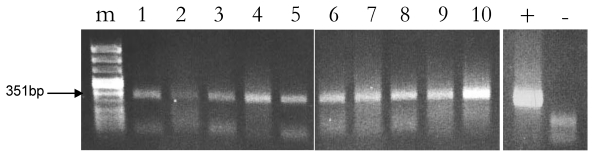
Detection of the 351 bp long rubisco fragment in total DNA extracted from raw cow milk samples of two different stock farms. Lane from 1 to 5: PCR reactions run on 400 ng of total DNA from plain-derived milk sample n° 9; lane from 6 to 10: PCR reactions on 400 ng of total DNA from mountain-derived milk sample n° 4. m: molecular marker; “+”: plant DNA PCR positive control; “-”: negative PCR control (just buffer).

One of the possible limitations of a PCR based method for DNA detection in milk is inhibition of Taq polymerase due to fat and protein components. As we have previously mentioned, a simple dilution of the sample may attenuate inhibition but it may also decrease test sensitivity when the DNA template is a limiting factor as it the case for the DNA of the diet. This may either result in false negatives or may cause variability of detection. Published data [18,19] report that milk protein content and fatty acid profile can be influenced by the dairy farming system, especially so when diets are consumed in different geographic area. To investigate about these aspects and to evaluate the level of reproducibility of our rubisco-based assay for plant DNA detection, *rbcL*-specific PCR-mediated amplifications were carried out on two raw cow milk samples collected from two distantly located stock farms, one set in the plain, the other in the mountain. PCR reactions were performed using the RUB-F2/R2 couple of primers, that amplified a 351 bp *rbcL* fragment. For each of the two milk lots, five PCR reactions were separately run on total DNA extracted from the skimmed fraction recovered after hexane-treatment. Sample n° 9 was from cows fed in a plain-located farm in Lombardy where animals were prevalently fed with commercial fodders. Milk sample n° 4 was instead collected in Valle d’Aosta, from a mountain-located farm where cows were mainly fed at pasture. These same milk samples were also analyzed and compared for the fatty acid profile. To this specific regard, milk sample n° 4 contains a lower percentage of saturated fatty acid compared to n° 9 (Secundo, personal communication). No substantial differences were instead observed in the level of *rbcL*-specific amplification, that was similarly successful for the two milk lots (Figure 3). We conclude that plant DNA amplification wasn’t influenced by the different fatty acid profile and the different provenance of milk. Similar experiments performed on brand-labeled milk sample n° 1 gave the same results: no differences between the multiple PCR replicates were ever observed (data not shown).

Some fluctuations in the intensity of the *rbcL*-specific amplification signal was actually observed when comparing the output of the PCR reactions. This indicates that, although successful, PCR-mediated plant DNA detection in milk needs further technical improvements. Statistically, even when we normalize for the same amount of total milk DNA, there is no clue that we are actually withdrawing the same amount of plant feed DNA that is present only in traces. This calls for technical improvements that can significantly augment the yield of extraction of available and reactive plant DNA sequences. 

### 2.4. Sequencing of Plant DNA Fragments Amplified in Cow Milk Samples

**Figure 4:**
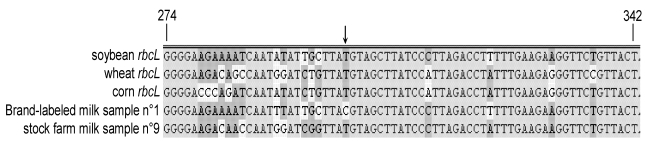
Multiple alignment of the *rbcL* sequence region comprised between nucleotide +274 trough +342 with respect to the ATG translational initiation codon, done by comparing the sequences of plant species commonly present in commercial fodders and the DNA rubisco fragments that have been amplified from n° 1 brand-labelled and n° 9 stock farm-collected cow milk samples. The arrow points to a nucleotide that is unique for the brand-labelled milk sample.

A further validation of our results came from DNA sequencing of the *rbcL* fragments amplified from total DNA of milk samples n° 4 and n° 9. The rubisco amplicon derived from total DNA extracted from n° 1 brand-labelled raw cow milk sample was also sequenced. The chromatogram profiles obtained from DNA sequencing reflected the different polyphytic composition of the diets, a likely consequence of the different dairy farming system. Consistently, when the *rbcL* sequences, amplified from either milk sample n° 9 or the commercially available milk were compared and analyzed with the NCBI/BLAST tools and VectorNTi software, SNPs corresponding to plant species commonly present in commercial fodders such as those of corn, wheat and soybean were identified (Figure 4). Remarkably, these are the components of the daily diet that is usually provided to animals grown in stock farms located in the plains of North Italy. It falls into this line that our DNA sequencing data have enabled us to predict the composition of the diet supplied to the cows that produce the brand-labelled milk sample. After consultation, the producer substantially validated our prediction. To this regard, the arrow in Figure 4 points to a nucleotide that is specific for the commercially distributed milk (sample n° 1) and is not attributable to any of the plant species mentioned above. Findings like this indicate the possibility that DNA sequencing may actually be used for the identification of uncertified components, undisclosed in the fodder tag, yet present in the animal feed. On the other hand, the *rbcL* sequences, retrieved from the amplification of the DNA extracted from milk sample n° 4 of mountain provenance, also presented some SNPs that could be specifically attributed to rubisco genes of different grasses as ascertained with the NCBI/BLAST tool (data not shown). 

Our results may raise some concern about the possibility that *rbcL* fragments amplification could actually occur because of accidental, environmental-mediated plant DNA contamination boosted up by the extreme sensitivity of the PCR technique. In addition to the fact that all possible precautions were taken to avoid any laboratory-derived plant DNA contamination (see experimental section), we exclude such a possibility since no positive signal for *rbcL* was ever registered in the PCR reactions run as negative controls (just buffer). This conclusion is further reinforced by the fact that only 70% of the analyzed milk samples were positive for plant DNA detection and the samples that were negative for such an amplification were nonetheless capable of sustaining the amplification of a 453 bp long β-*CN* fragment with the β-cas E7F/cas2R set of primers (data not shown). In addition, our DNA sequencing data seem to exclude any other possible source of contamination since the nucleotides composition reflects that of the plant species that were assumed with the diet and distinguishes milks produced in different locations. This latter could not be a trivial consequence since it may help in the identification of the actual site of milk production. In conclusion, these results on plant rubisco DNA provide the confidence that sequencing could be a valid tool for milk traceability. 

## 3. Experimental Section

### 3.1. Total Milk DNA Extraction

Three different protocols were at least twice performed for the total DNA extraction from different cow milk samples, both fetch in stock farm or sold in the Italian markets. For each extraction protocol tested, duplicate aliquot from each milk sample were taken.

For the CTAB-NaCl buffer precipitation method the extraction protocol according to what previously reported [12] with slight volume adjustment modifications was used. For the CTAB/phenol:chloroform extraction method, 20 mL of each milk sample was directly mixed with 60 mL CTAB buffer [1.4 M NaCl, 2% (wt/vol) CTAB (cetyltrimethylammonium bromide), 100 mM Tris, 15 mM EDTA, pH 8.0] and 240 μL of Proteinase K (20 mg/mL). The solution was kept overnight at 65 °C under constant agitation. 75 mL (vol/vol) of phenol: chloroform: iso-amyl-alcohol (25:24:1) were added to the supernatant samples and mixed gently. The aqueous phase was recovered after centrifugation for 15 min at 8,000 g. The extraction/centrifugation step was repeated twice. DNA was precipitated overnight at –20 °C with 1 vol of 2-propanol and 500 μL of glycogen (20 mg/mL). Then, samples were centrifugated at 8,000 g for 20 min at 4 °C. The pellet was washed twice with 75% (vol/vol) ethanol and dissolved in appropriated volume of double distilled sterile water. Samples were finally incubated for 30 min at 50 °C with 1/10 (v/v) of RNase A (100 mg/mL).

For the hexane extraction protocol, 1.2 mL of *n*-hexane was added to 20 mL of milk, mixed gently and centrifuged at 4 °C for 10 min at 8,000 *g*. During the centrifugation step at 4 °C a solid fat layer merge from supernatant allowing the removing with a sterile spatula. The skimmed fraction, about 17 mL, and the fat fraction, about 3 mL, so obtained were then extracted separately using the CTAB/phenol:chloroform extraction protocol mentioned above. 

Purity and quantity of the extracted DNA were determined with a spectrophotometer by measuring the absorbance at A_260_ and the A_260_/A_280_ ratio (DU^®^ 730 Beckmann Spectrophotometer). Aliquots of each DNA sample were also loaded on a 0.8% (wt/vol) agarose gel, and the DNA amount was further verified by comparison with known amounts of standard DNA (high molecular weight phage lambda DNA, M-Medical, Italy). 

### 3.2. PCR Methodology and Primer Design

Three sets of primers resulting in amplicons of different sizes were employed for the detection of the chloroplast target sequence of rubisco (*rbcL*) gene. The sequences of Arub fw/rv and the Lrub fw/rv sets of primers that amplify a 348 bp and 423 bp fragments respectively and the primer set RUB-F2/RUB-R2 that amplify a 351 bp fragment were previously described [11,15]. The β-cas E7F/cas2R primer set that amplify a 453 pb fragment for the β-*CN* gene amplification was also previously described [20]. All primers were purchased from PRIMM srl (Biomedical Science Park S.Raffaele, Milan, Italy).

To avoid any possible, laboratory-derived, plant DNA contamination PCR analyses done on DNA extracted from plant material were kept separate from those performed on milk-derived DNA samples. Lab sites, reagents and pipettes were different for the two procedures. In addition two controls for negative amplification (no DNA) were systematically added to every set of PCRs. PCR assays were replicated at least three times for each milk sample analyzed. Each PCR-based DNA amplification was performed in a total volume of 30 μL, in 1 × Taq Advanced buffer (Eppendorf), 2 mM MgCl_2_, 200 μM of each dNTP, 1 μM of each Primer and 1 U of Taq DNA Polymerase (Eppendorf). The amount/reaction of milk DNA was between 200 ng and 800 ng. The cycling conditions for each couple of primers are described below. PCR amplification was performed in a Eppendorf Mastercycler® Gradient thermal cycler. All PCR reactions were “hot started”: PCR tubes were placed in the PCR thermocycler machine when the block temperature reached 99 °C and then the normal temperature cycle profile was started as follow: 5 min at 95 °C for the initial denaturation, 45 cycles of amplification (30 s at 95 °C, 40 s at 60 °C, and 30 s at 72 °C), and 5 min at 72 °C for the final extension. The same amplification conditions, except for an annealing step performed for 90 s at 60 °C, were used with the β-casE7F/cas2R set of primers. DNA fragments amplified by PCR were loaded on a 1.5%–2% (wt/vol) agarose gels with suitable DNA markers. 

### 3.3. Sequence Analyses

To validate the positive PCR results, PCR products from 3 different milk samples were gel purified using the QIAquick Gel Extraction Kit (Qiagen) according to the manufacturer's instructions and then sequenced at PRIMM srl (Biomedical Science Park S.Raffaele, Milan, Italy). The nucleotide sequences of the *rbcL* fragments were analyzed using the tBLASTn facility http://www.ncbi.nlm.nih.gov/BLAST/ and the VectorNTi AlignX program (Invitrogen) 

## 4. Conclusions

We have shown that feed-derived plant DNA fragments can be detected in raw cow milk samples with the use of PCR-based assays. Detection was equally successful in milk samples fetched from stock farms or sold in the Italian market. These PCR techniques, in combination with DNA sequencing, may prove useful for food surveillance services. Plant DNA detection in milk is relying on the use of chloroplast-based gene sequences such as that of *rbcL*. This is a mandatory option since chloroplast-based gene sequences are in high copy number/cell and that makes detection of rare plant DNA fragments a feasible task. In addition, the chloroplast genome of many plant species commonly used for animal feeding is completely sequenced. This should allow an easy search for new additional chloroplast-based DNA sequences that contain a higher percentage of SNPs substitution compared to what we found for the *rbcL* genes. SNPs can be very helpful to design new protocols for DNA amplification that can lead to the concomitant identification of the different plant species present in the diet. The unambiguous characterization and correlation of what is amplified in milk and what was present in the commercial fodder or in a specific pasture area could lead to the development of new tools for traceability and/or authenticity purposes specific for the food chain of dairy products. These improvements open the possibility that, in the future, many type of cheeses requiring the Protected Designation of Origin (PDO) certification could be validated by the search of the plastidial DNA fragments present in the milk used for their production.
